# A novel model for hemolysis estimation in rotating impeller blood pumps considering red blood cell aging

**DOI:** 10.3389/fphys.2023.1174188

**Published:** 2023-04-12

**Authors:** Liang Wang, Zhong Yun, Jinfu Yao, Xiaoyan Tang, Yunhao Feng, Chuang Xiang

**Affiliations:** ^1^ School of Mechanical and Electrical Engineering, Central South University, Changsha, China; ^2^ College of Mechanical Engineering, Hunan University of Arts and Science, Changde, China

**Keywords:** red blood cell aging, rotating impeller blood pump, shear injury, hemolysis estimation, optimization

## Abstract

For blood pumps with a rotating vane-structure, hemolysis values are estimated using a stress-based power-law model. It has been reported that this method does not consider the red blood cell (RBC) membrane’s shear resistance, leading to inaccurate estimation of the hemolysis value. The focus of this study was to propose a novel hemolysis model which can more accurately predict the hemolysis value when designing the axial flow blood pump. The movement behavior of a single RBC in the shear flow field was simulated at the mesoscale. The critical value of shear stress for physiological injury of RBCs was determined. According to the critical value, the equivalent treatment of RBC aging was studied. A novel hemolysis model was established considering the RBC’s aging and the hemolysis’ initial value. The model’s validity was verified under the experimental conditions of shear stress loading and the conditions of the shear flow field of the blood pump. The results showed that compared with other hemolysis models for estimating the hemolysis value of blood pumps, the novel hemolysis model proposed in this paper could effectively reduce the estimation error of the hemolysis value and provide a reference for the optimal design of rotary vane blood pumps.

## 1 Introduction

In recent years, the increase in the number of patients with heart failure and the acute shortage of heart transplant donors have led to the gradual replacement of ventricular assist devices (VADs, also known as blood pumps) with heart transplantation as an effective treatment for heart failure (Bellumkonda and Bonde, 2012; Molina and Boyce, 2013; Magruder et al., 2017; Gaffey et al., 2018; Grace et al., 2018; Kawabori et al., 2018; Muhammad et al., 2018; Wu et al., 2021).

However, for medical devices that come into contact with blood, mechanical damage to blood is the focus of research prior to clinical application of medical devices. In particular, the shear stress generated by the high-speed rotating impeller of the vane blood pump, which exposes red blood cells in blood to non-physiological shear damage that is much higher than that of physiological shear damage (Tie-yan et al., 2013; Faghih and Sharp, 2019). In order to enhance the hemolysis performance of blood pumps, several studies have been proposed to optimize the design of blood pumps with the goal of hemolysis performance and to establish a hemolysis model (Zimmer et al., 2000; Lim et al., 2001; Chan et al., 2002; Grigioni et al., 2005; Goubergrits, 2006; Taskin et al., 2012) to estimate the hemolysis performance of blood pumps during the optimization design process. However, for complex flow fields inside rotating impeller blood pumps, the existing hemolysis model estimates are sufficiently inaccurate and have large errors compared to the reported hemolysis experiments (Wurzinger, 1986; Paul et al., 1999; Faghih and Sharp, 2019).

This study aimed to obtain the equivalent shear stress of RBC aging by the threshold model and establish a novel model for hemolysis estimation based on the power-law formula hemolysis model. The novel hemolysis model could provide a more accurate prediction for the optimal design of rotary vane blood pumps. First, the motion behavior of individual RBCs in the shear flow field was simulated at the mesoscale, and the critical value of the physiological damage shear stress of RBCs was determined. Second, a novel model for hemolysis estimation was developed by adding the aging equivalence and the initial value of hemolysis. Finally, the validity of the novel model for hemolysis estimation was verified under the experimental conditions of shear loading and the conditions of the shear flow field in the blood pump.

## 2 Materials and methods

### 2.1 Power-law hemolysis model

Most of the current research studies reported on the quantitative estimation of hemolysis models were based on the hemolysis model proposed by Giersiepen (Giersiepen et al., 1990). Giersiepen defined the hemolysis index ( HI ) as the ratio of the increment of plasma-free hemoglobin concentration ( ∆Hb ) to the total hemoglobin concentration in the blood ( Hb ), as a power function of shear stress and the exposure time of RBCs under shear stress, which is expressed as follows (Wu et al., 2022):

$$HI=\frac{∆Hb}{Hb}\times 100=C{∙\tau }^{\alpha }{∙t}_{\exp}^{\beta },$$ (1)

where *τ* represents the shear stress (Pa); *t* represents the exposure time (s); and *C*, *α*, and *β* are constants, $C=3.62\times 10^{-5}$ , α=2.416 , and β=0.875 . They are obtained after regression analysis based on the experimental data of Wurzinger (1986).

**FIGURE 1:**
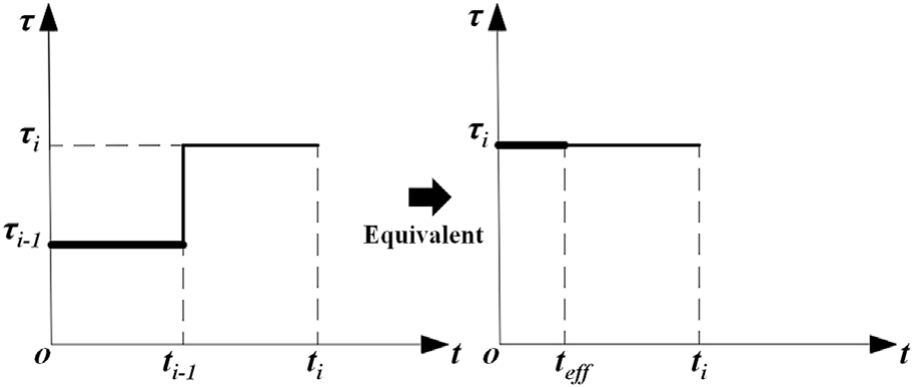
Schematic illustration of the imaginary time equivalence principle.

To predict hemolysis more accurately, Goubergrits and Affeld (2004) considered the loading history of shear stress and defined the virtual time $t_{eff}$ as an intermediate variable, i.e., the time required to produce the same hemolysis value under shear stress $\tau_{i}$ as the time period during which shear stress $\tau_{i-1}$ acts as $t_{i-1}$ . Figure 1 shows the equivalence principle when computing the imaginary time.

Then, the expression for the imaginary time is computed as follows:

$$t_{eff}={\left[\frac{HI\left(t_{i-1}\right)}{C∙\tau_{i}^{\alpha }}\right]}^{\frac{1}{\beta }}={\left[\frac{\left.\tau_{i-1}^{\alpha }∙{\left(t_{i-1}\right)}^{\beta }\right)}{\tau_{i}^{\alpha }}\right]}^{\frac{1}{\beta }}.$$ (2)

The expression of the hemolysis model is

$$\left\{\begin{array}{c} {HI}_{eff}\left(t_{i}\right)=C∙\tau_{i}^{\alpha }∙{\left[t_{eff}+\left(t_{i}-t_{i-1}\right)\right]}^{\beta },\\ t_{eff}={\left[\frac{\tau_{i-1}^{\alpha }∙t_{i-1}^{\beta })}{\tau_{i}^{\alpha }}\right]}^{\frac{1}{\beta }}. \end{array}\right.$$ (3)

Although the shear stress loading history could effectively reduce the hemolysis estimation error, the RBCs still had a specific shear resistance, and hemolysis did not occur when the shear stress was less than a certain threshold value. So, it was not reasonable to directly use the power-law formula to estimate the hemolysis value.

### 2.2 RBC aging-equivalent shear stress

For the study of the relationship between exposure time and critical shear stress, Eilers (1997) concluded that for a specific exposure time $t_{\exp}$ , no hemolysis occurred when the shear force was less than $\tau_{crit}$ . An empirical formula for the critical shear force $\tau_{crit}$ , also known as the threshold formula, is proposed as follows:

$$\tau_{crit}=88.905t_{\exp}^{-0.3372}.$$ (4)

The aging process of RBCs, which began with the release of RBCs from the bone marrow into the circulation and ended with destruction, fluctuated in the duration from 70 to 140 days, with an average of 115 days (Cohen et al., 2008; Mock et al., 2011). From the hemolysis perspective, the RBCs’ value reached 100% after 115 days in the circulatory system, and the threshold model allowed us to obtain the average equivalent shear stress to which RBCs were subjected during their life cycle as 0.3886 Pa.

### 2.3 Analysis of the motion behavior of RBCs in shear flow fields

Chen et al. (2016) and Chen et al. (2017) reported that scalar shear stresses within the blood pump were classified into three categories: below 25 Pa was in the normal physiological range, between 25 and 125 Pa was the elevated non-physiological range, and above 125 Pa was the highly elevated non-physiological shear stress range that might lead to RBC destruction. During their lifetime, RBCs had the property of returning to their original state after passing through capillaries that are much smaller in diameter than themselves. This property indicates that the RBCs could withstand some shear stress without mechanical damage.

Thus, the damage process at shear stresses below 25 Pa could be considered an aging problem of the RBCs. At shear loads greater than 25 Pa, it was a non-physiological shear range that caused mechanical damage to the RBCs and caused hemolysis.

For the study of the pump flow field at the shear-damaging threshold of RBCs, the results obtained for the shear threshold of RBCs differ due to the different experimental methods used, parameters of the theoretical calculations, and other factors. There were 255 Pa (Shi et al., 2014), 400 Pa (Pan et al., 2011), 600 Pa (Grigioni et al., 2005), and 800 Pa ranges (Lu et al., 2001), and the corresponding exposure times were 1 ms, 700 ms, 4 min, and 1 ms, respectively. We studied the morphology, perimeter, and total elastic strain energy of the RBCs in the shear flow field according to the shear threshold and physiological shear damage threshold of the RBCs reported previously.

A single RBC shear flow field environment was built, mainly through two infinite extensions of the parallel plate; the spacing was δ , the fluid between the plates had a certain viscosity μ , the lower plate was fixed, and the upper plate with speed v was to the right side. Then, the shear flow field could be formed between the two plates. The relationship between the upper plate moving speed and the shear stress τ between the two plates can be expressed as

$$v=\frac{\tau ∙\delta }{\mu }.$$ (5)

It has been reported that RBCs subjected to a certain degree of shear stress undergo a certain amount of deformation, and when the shear stress ceases to act, the RBCs return to a biconcave pancake shape, whose biconcave pancake shape can be expressed as follows (Lanlan. et al., 2013):

$$z=\pm D_{0}\sqrt{1-\frac{4\left(x^{2}+y^{2}\right)}{D_{0}^{2}}}∙\left[a_{0}+a_{1}\frac{x^{2}+y^{2}}{D_{0}}+a_{2}\frac{{\left(x^{2}+y^{2}\right)}^{2}}{D_{0}^{4}}\right],$$ (6)

where $D_{0}$ represents the RBC diameter and takes the value of 7.82 μm; $a_{0}$ , $a_{1}$ , and $a_{2}$ represent the RBC thickness descriptive parameters and take the values of 0.0518, 2.0026, and −4.491, respectively.

The fluid flow in the narrow gap causes the RBCs to move and deform, and the deformation produced by the RBCs simultaneously acts back on the fluid. At this point, the action between the RBC membrane and the fluid is strongly coupled. The strong coupling will significantly enhance the non-linearity of the model. Therefore, the flow–solid coupling approach was used to model the RBCs, and for the blood flow in the blood pump, the blood was considered an incompressible Newtonian fluid. The hydrodynamic equations for blood are

$$\rho_{fluid}\left[\frac{\partial U_{fluid}}{\partial t}+\left(U_{fluid}∙\nabla \right)U_{fluid}\right]=\nabla \left[\mu \nabla U_{fluid}+\mu {\left(\nabla U_{fluid}\right)}^{T}\right]+F,$$

$$\nabla U_{fluid}=0,$$

where $\rho_{fluid}$ represents the fluid density, $U_{fluid}$ represents the velocity vector of the fluid, μ represents the hydrodynamic viscosity, F represents the external force vector, and ∇ corresponds to ${\left(\partial /\partial x,\partial /\partial y\right)}^{T}$ .

The RBC membrane was treated as an isotropic viscoelastic membrane, and the equations of motion are expressed as

$$\rho_{solid}\frac{\partial^{2}u_{solid}}{\partial t^{2}}=\nabla \sigma +F_{V},$$

where $\rho_{solid}$ represents the density of the RBC membrane, $u_{solid}$ represents the displacement vector of the RBC membrane, σ represents the stress tensor of the RBC membrane, and $F_{V}$ represents the volume force vector on RBCs.

Due to the viscoelastic nature of the RBC membrane, the viscoelastic behavior of RBC was described using the Kelvin–Voigt model with the following relationship between the elastic strain rate and stress:

$$\sigma =2\eta \frac{d\varepsilon }{dt},$$

where η represents the viscosity of the RBC membrane; ε represents the strain tensor of the RBC membrane and is expressed as follows:

$$\varepsilon \left(t\right)=\frac{\sigma_{0}}{G}\left(1-e^{-\frac{t}{\tau }}\right).$$

Here, $\sigma_{0}$ represents the initial stress tensor of the RBC membrane; τ represents the relaxation time, obtained from the ratio between the viscosity of the film and the shear modulus; G represents the shear modulus of the RBC membrane; and τ and G are expressed as

$$\tau =\frac{\eta }{G},$$

$$G=\frac{E}{2\left(1+v\right)}.$$

Here, E and v represent the Young’s modulus and Poisson’s ratio of the RBC membrane, respectively.

The interaction at the fluid–solid interface was due to the fluid load on the solid and the displacement of the solid within the flowing fluid, which could be expressed as

σn=Γn,

$$U_{fluid}=v_{w},$$

$$v_{w}=\frac{\partial u_{solid}}{\partial t},$$

where Γ represents the total force exerted on the solid boundary by the fluid, n represents the unit normal vector of the fluid–solid interface, and $v_{w}$ represents the velocity vector of the movement of the RBC membrane.

### 2.4 A novel model for hemolysis estimation

(1) Initial values of hemolysis

Yeleswarapu et al. (1995) detected an increase in plasma-free hemoglobin concentration, ∆*PfHb* = 69.3 mg%, after blood was exposed to a constant shear stress of 700 pa for 25 ms, and this shear stress and exposure time were used to calculate the hemolysis value, HI = 14.957%, according to Eq. 1.

Li et al. (2015) used a coefficient k to describe the relationship between the detected increase in hemoglobin concentration ( ∆PfHb ) and the hemolysis value ( HI ) estimated by the hemolysis model to enable a cross-sectional comparison of experimental data.

$$k=\frac{∆PfHb\left(mg\%\right)}{HI\left(\%\right)}=4.63.$$

Yeleswarapu et al. detected that the initial value of hemoglobin concentration ( ${PfHb}_{0}$ ) was 25.47 mg/dL in the hemolysis experiment under time-varying shear stress, which indicated that a certain degree of hemolysis had occurred before the experiment began. For RBCs that did not fully rupture, their shear resistance might also be affected. At this point, the effect of the initial hemolysis value on the estimated hemolysis value should not be neglected. Because the RBCs in the blood were continually updated, each RBC in the blood was at different life values at a certain time, but the overall hemoglobin concentration in normal human blood was roughly stable, and the initial hemolysis value ( ${HI}_{0}$ ) of the blood would be obtained by using the coefficient k , which is expressed as

$${HI}_{0}=\frac{{PfHb}_{0}}{k}=5.5\%.$$

(2) A novel model for hemolysis estimation

**FIGURE 2:**
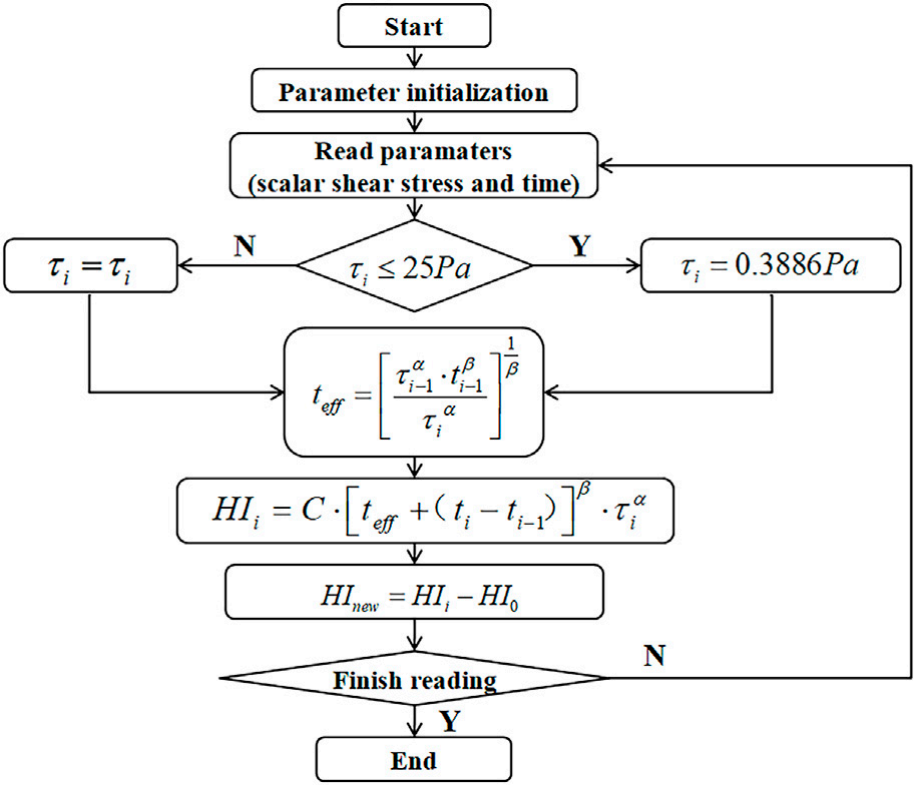
Novel model hemolysis prognosis process.

In summary, considering the shear loading history, the equivalent shear stress of aging RBCs, and the physiological shear threshold of RBCs, a novel hemolytic prognosis model based on the aforementioned hemolytic Eq. 3 was developed, and the novel model hemolytic prognosis flow is shown in Figure 2.

Thus, a novel model of hemolysis considering RBC aging is as follows:

$$\left\{\begin{array}{c} \tau_{i}=0.3886Pa\left(\tau_{i}\leq 25Pa\right),\\ {HI}_{0}=5.5\%,\\ {HI}_{i}=C∙{\left[t_{eff}+\left(t_{i}-t_{i-1}\right)\right]}^{\beta }∙\tau_{i}^{\alpha },\\ t_{eff}={\left[\frac{\tau_{i-1}^{\alpha }∙t_{i-1}^{\beta }}{\tau_{i}^{\alpha }}\right]}^{\frac{1}{\beta }},\\ {HI}_{new}={HI}_{i}-{HI}_{0}. \end{array}\right.$$ (7)

The values of empirical coefficient parameters C , α , and β are 3.62 × 10^−5^, 2.416, and 0.785, respectively.

### 2.5 Model validation methods

In computational fluid dynamics (CFD), the Lagrange particle tracing method could be used to obtain the shear stress tensor of RBCs on the flow field trace in the pump. However, because the flow inside the blood pump was a three-dimensional complex flow, it was necessary to convert the shear stress tensor reading in the flow field into a scalar shear stress and then bring it into the hemolysis model to solve the hemolysis value. The expression of the flow field shear stress tensor is

$$\tau_{ij}=\eta \frac{\partial v_{i}}{\partial x_{j}},$$ (8)

where η represents the blood viscosity. Bludszuweit (1995) formulation of equivalent shear stress based on the Mises’ yield criterion is widely used and expressed as

$$\tau_{s}={\left[\frac{1}{6}\sum {\left(\tau_{ii}-\tau_{jj}\right)}^{2}+\sum \tau_{ij}^{2}\right]}^{\frac{1}{2}}.$$ (9)

The best way to validate the hemolysis model would be to use a hemolysis experiment with a loaded shear stress, but this was not possible due to the difficulty in accurately controlling the blood variable shear stress of the experimental setup and the fact that there were few relevant measured reference data for hemolysis experiments. Therefore, this paper used the new model and other models to estimate hemolysis under different working conditions. The comparison verified the validity of the new model.

(1) Shear stress loading experimental conditions

**FIGURE 3:**
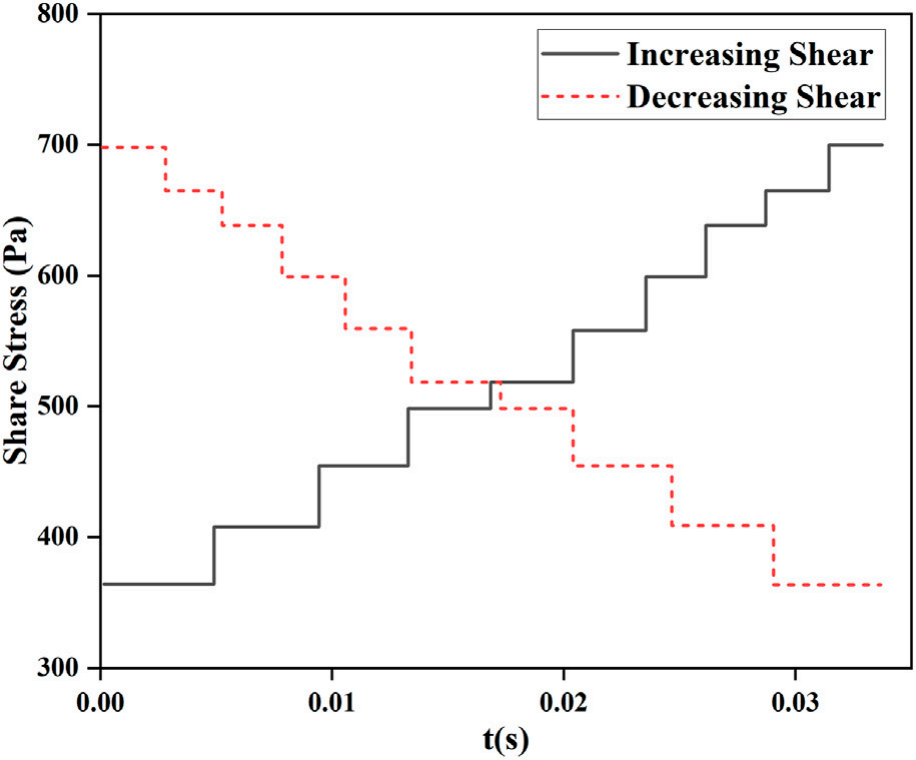
Variation in loaded shear stress with time studied by Yeleswarapu et al. (1995).

For the validation of the novel hemolysis model under the experimental conditions of shear loading, the experimental data from Yeleswarapu et al. on shear loading were used and combined with the estimated values of the hemolytic model for comparative validation. During the experiments of Yeleswarapu et al. to study the shear stress on blood damage, two conditions of decreasing and increasing shear stress with time were loaded. The shear stress loading with time is shown in Figure 3.

(2) Axial flow pump shear flow field conditions

**FIGURE 4:**
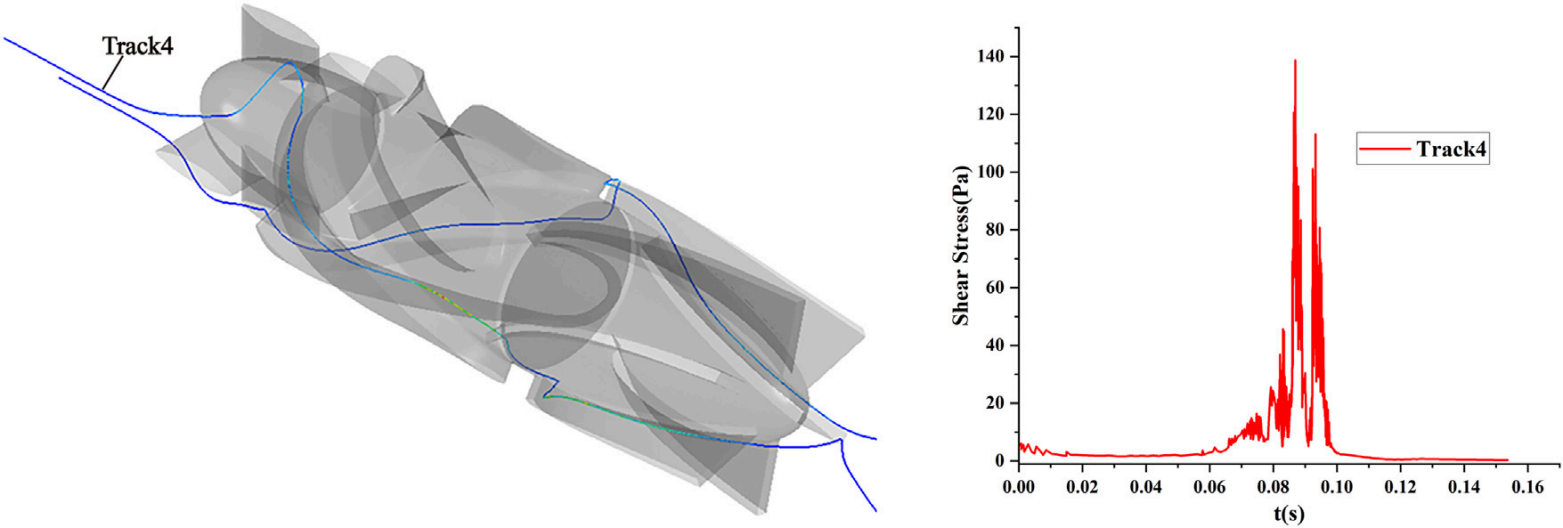
Scalar shear stress and exposure time of the particle traces for number 4 under the shear flow field condition of the axial flow pump.

Particles representing RBCs were released through the inlet of the blood pump, and the particles were traced using the Lagrange method. The statistical shear stress and exposure time on the traces could be used for blood pump hemolysis estimation. The shear stress and exposure time on the traces of one of the particles numbered 4 are shown in Figure 4.

**FIGURE 5:**
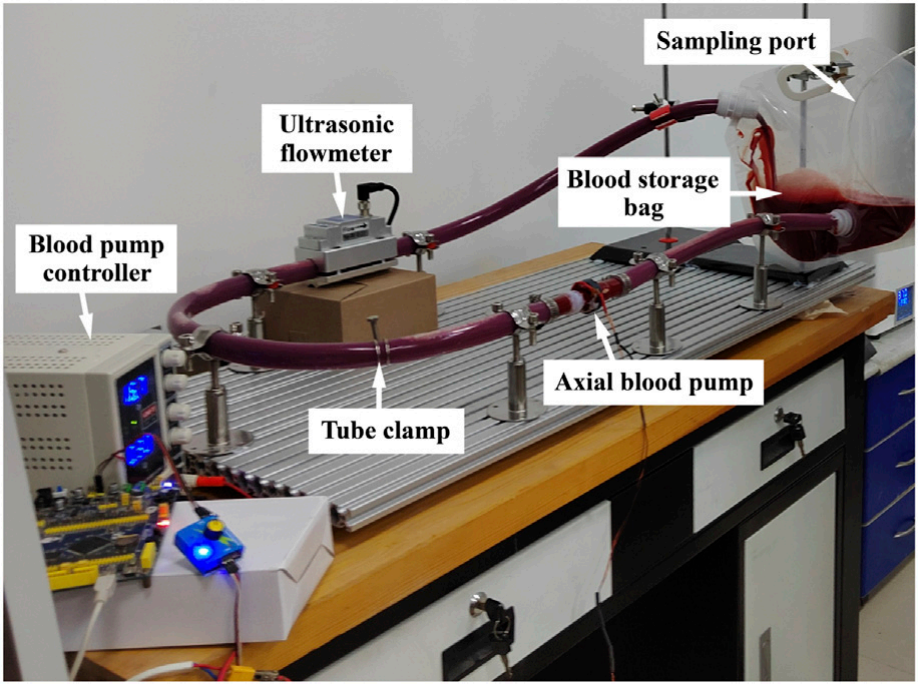
Hemolysis test circulation platform.

In order to verify the validity of the hemolysis estimation for the blood pump, a hemolysis test study was conducted on a magneto-hydraulic suspended axial flow pump, and the blood circulation platform built is shown in Figure 5. Fresh porcine blood was used as a blood sample in the experimental circulatory tubing circuit. The normal range of the total hemoglobin concentration in porcine blood was 90–130 g/L, and the normal range of hematocrit was 32%–43%. Fresh porcine blood obtained from a local slaughterhouse was anticoagulated with 3,000 USP of heparin per 500 mL of blood. Porcine blood should be stored under refrigeration at 4°C for no longer than 48 h. Before the experiment, PBS solution was injected into the circulatory tubing circuit, which was circulated and flushed for 15 min, after which PBS was drained. A measure of 1 L of anticoagulated porcine blood was injected into the circulatory tubing circuit, the blood pump speed was set to about 8,000 r/min to achieve blood pumping circulation, and the ambient temperature of the laboratory was 25°C–26°C. From the start of pumping, samples were taken from the sampling port every 30 min, and the total circulation time of the experiment was 5.5 h. The hemolysis value was analyzed according to the free hemoglobin measurement.

In this paper, the plasma-free hemoglobin content was determined by using the colorimetric method. The micro-free hemoglobin (*freeHb*) content assay kit A071 (developed by the Nanjing Jiancheng Institute of Biological Engineering) was used for the analysis. A linear regression analysis fitted the measured plasma-free hemoglobin concentration (*freeHb*) data into a straight line. The slope of the line was the increment of the plasma-free hemoglobin concentration (*∆freeHb*) during the experimental sampling interval. The formula for calculating the standardized hemolysis index NIH based on the measured incremental plasma-free hemoglobin concentration is (Tie-yan et al., 2013)

$$NIH\left(g/L\right)=∆freeHb\times V\times \frac{100-Ht}{100}\times \frac{100}{Q\times T},$$ (10)

where ∆freeHb represents the increment of the plasma-free hemoglobin concentration (g/L) during the sampling interval; Ht represents the RBC pressure volume (%), 37; V represents the volume of circulating blood (L), 1 L; Q represents the flow rate (L/min), 5 L/min; and T represents the sampling time interval (min), 30 min.

To compare the estimated hemolysis value with the experimental hemolysis value, the relationship between *NIH* and *HI* was obtained according to the additional descriptive form of *NIH* (Carswell et al., 2013) and combined with Eq. 1; then, the formula for the experimental hemolysis value is expressed as

$${HI}_{\mathrm{Exp}}\left(\%\right)=\frac{∆Hb}{Hb}\times 100=\frac{NIH\left(g/100L\right)\times 100}{\left(100-H_{t}\right)∙\kappa },$$ (11)

where κ represents the amount of hemoglobin in the blood and is generally taken as 110 g/L.

## 3 Results and discussion

### 3.1 Results of the simulation of RBC motility in shear flow fields

Generating shear flow fields of different strengths required the moving speed of the upper plate to be calculated according to Eq. 5, and additional parameter settings are shown in Table 1.

**TABLE 1:** Shear flow field setting parameters.

Parameter	Young’s modulus of the membrane	Poisson’s ratio of the membrane	Dynamic viscosity of the membrane	Density of the membrane	Density of the intracellular medium	Density of the intracellular medium	Dynamic viscosity of the extracellular medium	Density of the extracellular medium
Value	500 Pa	0.45	0.022 Pa s	1,090 kg/m^3^	1,060 kg/m^3^	0.006 Pa s	0.0012 Pa s	1,060 kg/m^3^

#### 3.1.1 Changes in RBC morphology under different shear stresses

**FIGURE 6:**
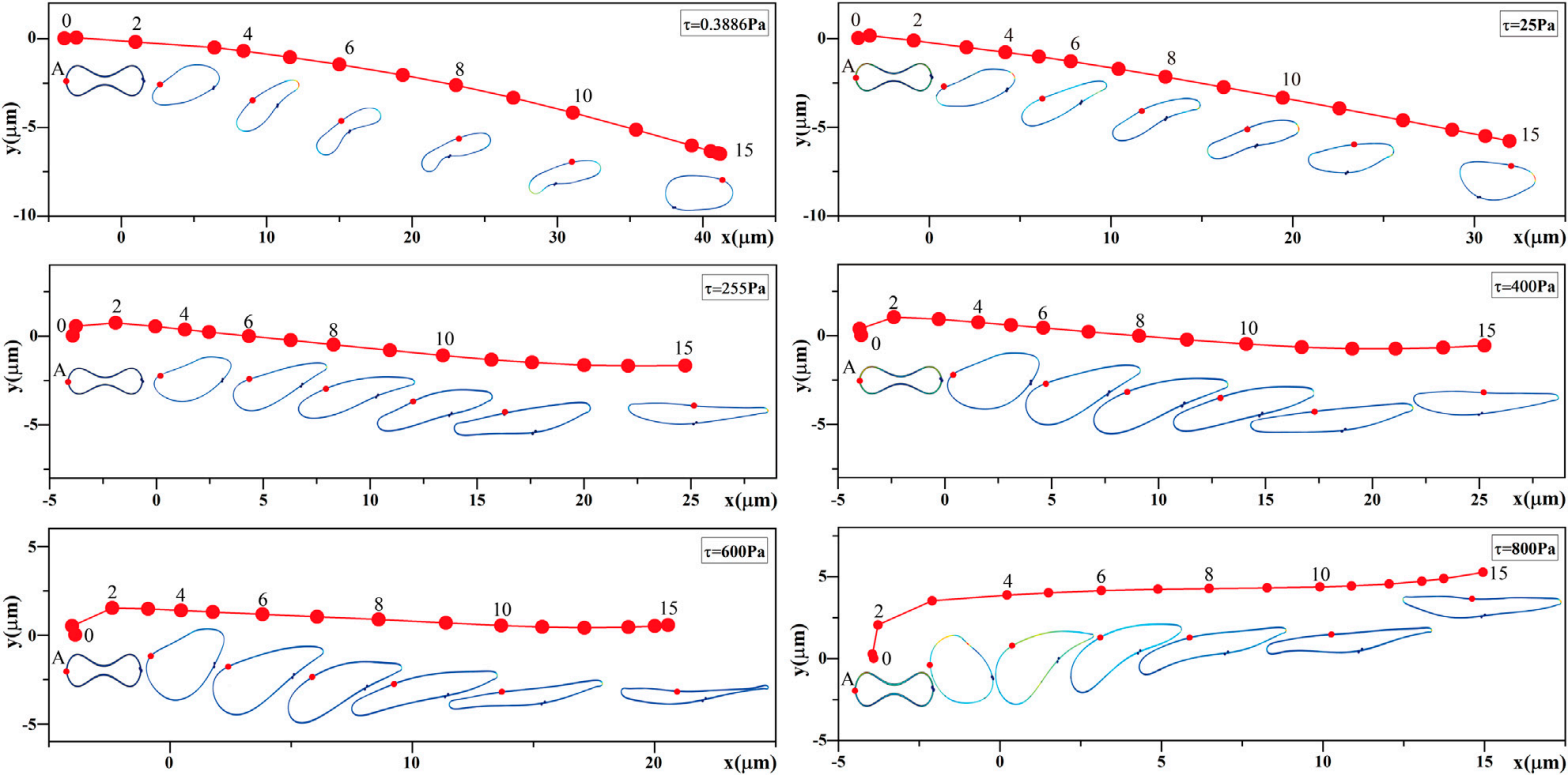
Changes in the RBC motility and morphology.

Point A at the left end of the long axis in the initial state of the RBC is set as the marker point, and the trajectory of point A and the morphology of the RBC at some nodes under the corresponding shear stress are shown in Figure 6.

Figure 6 shows that: (1) RBCs lost their biconcave pancake-like shape characteristics when shear stress was applied, and the overall main motion direction of RBCs was the same as that of the upper plate. (2) From the position of point A relative to the cell membrane, there was a rotational motion of the RBC membrane around its center of mass, and the speed of the rotational motion was greater when the shear stress was greater. (3) In the shear flow field, when the shear force was small, the RBCs moved in the negative direction of the *Y*-axis, and the trajectory was parabolic. As the shear force increased, the distance of RBCs moving in the negative direction of the *Y*-axis became smaller, and the parabola became flat. When the shear force continued to increase above 600 Pa, the RBCs moved in the positive direction of the *Y*-axis. (4) The comparative analysis showed that when the shear stress in the shear flow field was 25 Pa or below, the RBC stretching changes were not obvious.

#### 3.1.2 Changes in RBC perimeter under different shear stresses

**FIGURE 7:**
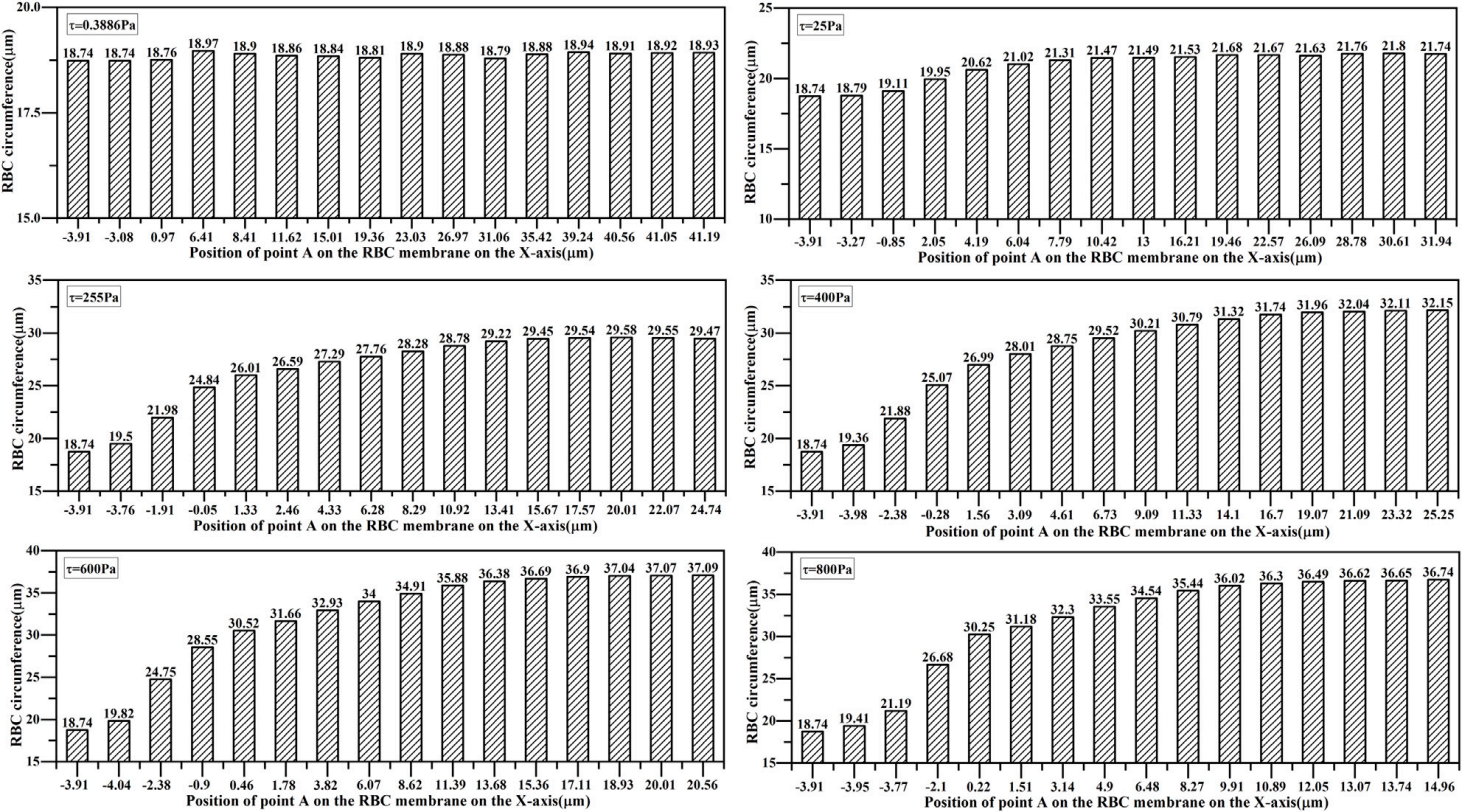
Changes in the circumference of RBCs.

Studies of RBC deformation typically used the ratio of the major and minor axes defined as the deformation index, whereas the deformation of RBCs in a shear flow field was complex and unreliable to describe using the deformation index. Therefore, the change in RBC perimeter could be better than the deformation and stretching of the cell membrane of RBCs subjected to the shear state, and the change in RBC perimeter in shear flow fields of different intensities is shown in Figure 7.

Figure 7 shows that: (1) the perimeter of RBCs increased with the increasing shear stress, which was consistent with the trend of the morphological changes shown in Figure 6. (2) When the shear stress was 0.3886 Pa, the maximum stretching length of RBCs is 1.012 times the original perimeter, indicating that the change in RBC girth was negligible, and it was reasonable to take 0.3886 Pa as the equivalent shear stress of RBC aging. (3) When the shear stress was 25 Pa, the maximum stretching length of RBCs was 1.16 times the original perimeter; when the shear stress force reached 400 Pa, the maximum stretch length of the RBCs was 1.72 times the original perimeter.

#### 3.1.3 Changes in the total elastic strain energy of RBCs under different shear stresses

**FIGURE 8:**
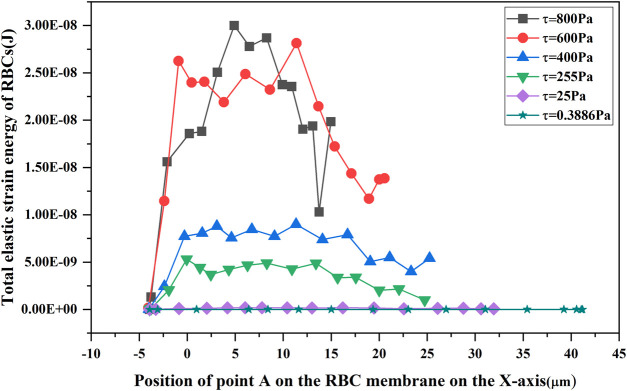
Changes in the total elastic strain energy of the RBCs.

The total elastic strain energy changes in RBCs under different shear stresses are shown in Figure 8. The maximum value of the total elastic strain energy of RBCs increased with the increasing shear stress. The changes in the total elastic strain energy of RBCs under 600 and 800 Pa shear stresses were similar, and the changes in the total elastic strain energy of RBCs in the shear flow fields below 400 Pa were considerably smaller than those in the shear flow fields above 600 Pa. Moreover, the total RBC elastic energy was nearly the same in 25 and 0.3886 Pa shear flow fields.

A comprehensive analysis of morphological changes, perimeter changes, and total elastic strain energy changes in RBCs in shear flow fields of different intensities showed that it was reasonable and reliable to treat RBCs subjected to shear stress below 25 Pa as equivalent to aging for hemolytic prognosis model studies.

### 3.2 Validation results of a novel model for hemolysis estimation

#### 3.2.1 Comparison of the hemolysis results under shear stress loading experimental conditions

**FIGURE 9:**
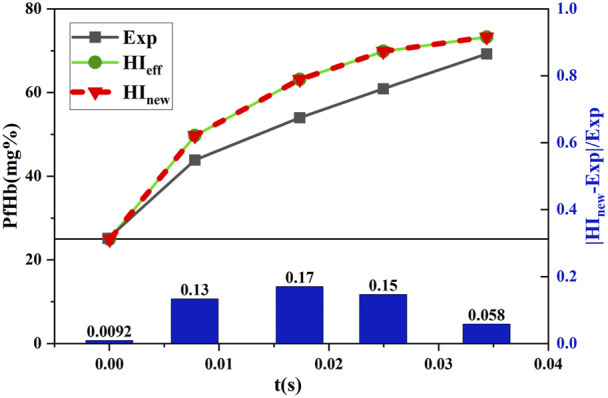
Comparison between the estimated and experimental hemolysis values with the decreasing shear force under time working conditions.

**FIGURE 10:**
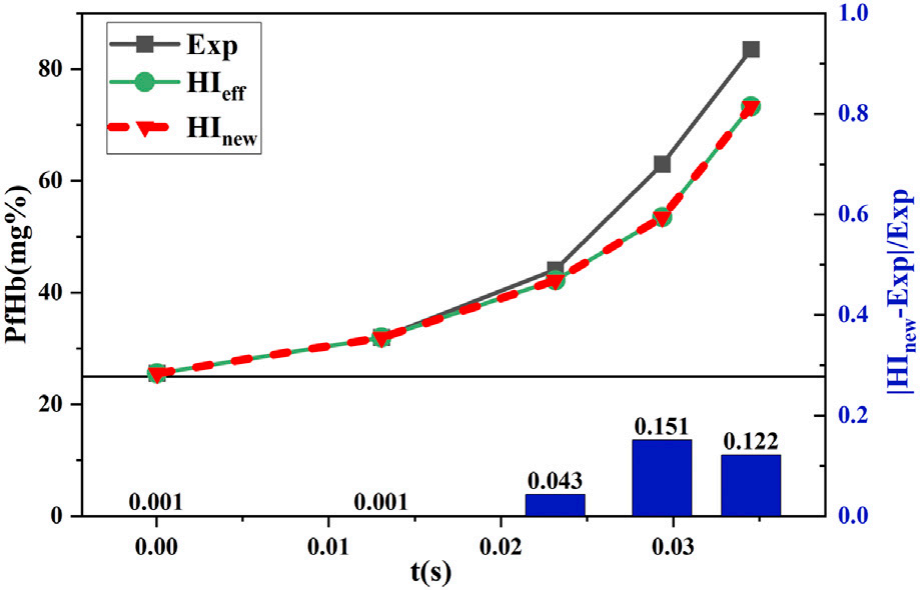
Comparison between the estimated and experimental hemolysis values with the increasing shear force under time working conditions.

The shear stress loading with time data, shown in Figure 3, was substituted into the hemolysis model of Eq. 3 and Eq. 7 to obtain the hemolysis estimates ${HI}_{eff}$ and ${HI}_{new}$ , which were then converted to hemoglobin concentration values by the factor k=4.63 and compared with the experimental values (Exp) of Yeleswarapu et al. The data obtained for the condition where the shear stress decreased with time are shown in Figure 9, and the data obtained for the condition where the shear stress increased with time are shown in Figure 10.

Figure 9 and Figure 10 show that ${HI}_{eff}$ and ${HI}_{new}$ coincide. This was because the experimental conditions for shear stress loading were unavailable for shear forces below 25 Pa, and the equivalent aging treatment of the new model was invalid. Moreover, the initial values of hemolysis were provided in this condition. Therefore, Eq. 7 was equivalent to Eq. 3. Figure 9 shows that the relative error between the estimated and experimental hemolysis value reached a maximum of 17% for shear stress decreasing with the time conditions which further decreased to 5.8% by the end of shear stress loading. Figure 10 shows that the relative error between the estimated and experimental hemolysis values reached a maximum of 15.1% with increasing shear stress with the time which further decreased to 12.2% by the end of shear stress loading. This indicated that the novel hemolysis model was plausible for estimating hemolysis under operating conditions without shear stress below 25 Pa.

#### 3.2.2 Estimation and experimental comparison of hemolysis under shear flow field conditions in axial flow pumps

**FIGURE 11:**
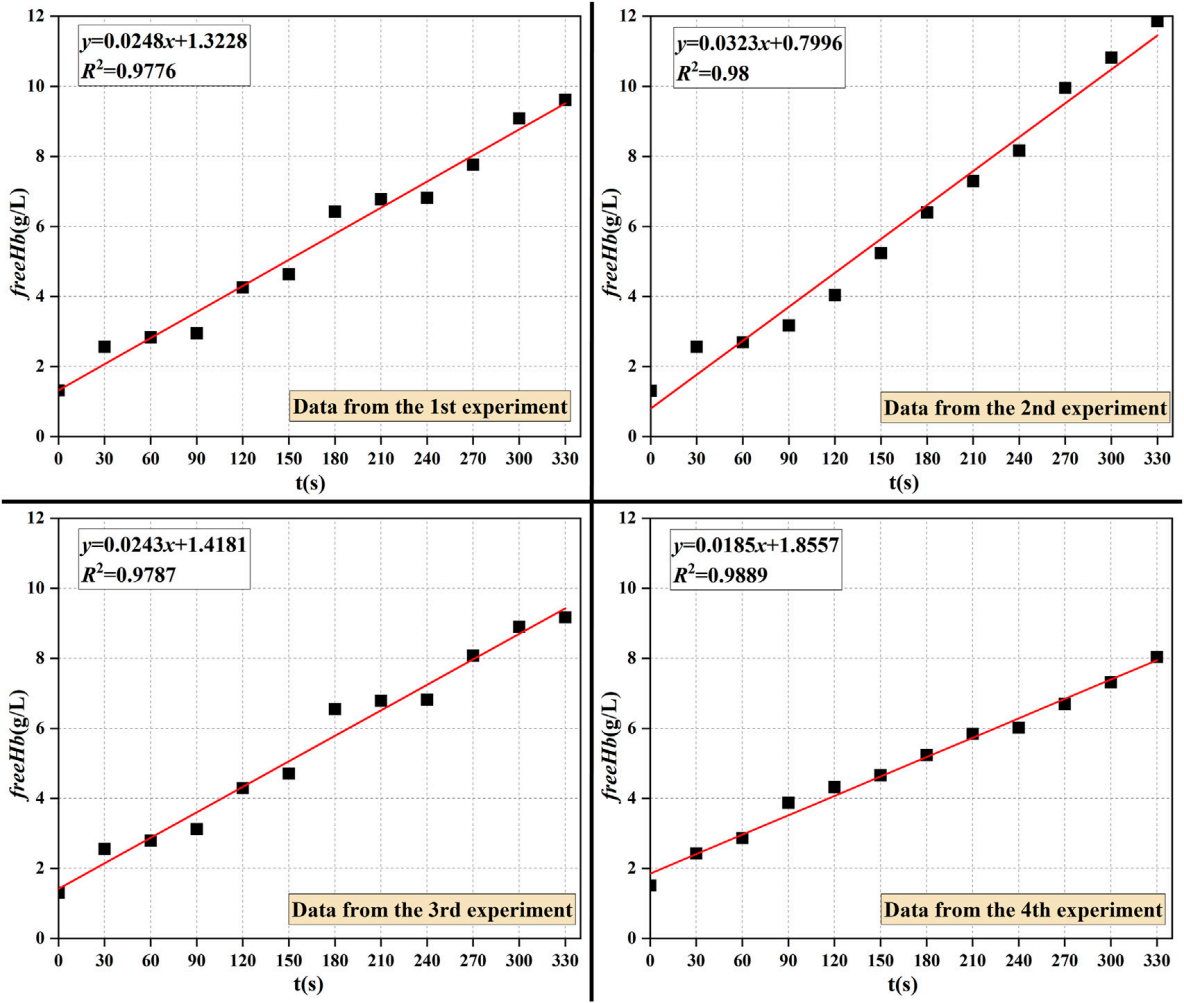
Experimentally measured plasma-free protein concentration over time.

The measured data and data processing results after 5.5 h of *in vitro* hemolysis experiments (*n* = 4) with the 3D-printed blood pump are shown in Figure 11. The standardized hemolysis index *NIH* was calculated by substituting ∆*freeHb* obtained from each experiment into Eq. 10. Then, the *NIH* value was substituted into Eq. 11 to get the hemolysis value ( ${HI}_{\exp}=0.0001515\pm 0.000034\%$ ) under the experimental conditions.

**FIGURE 12:**
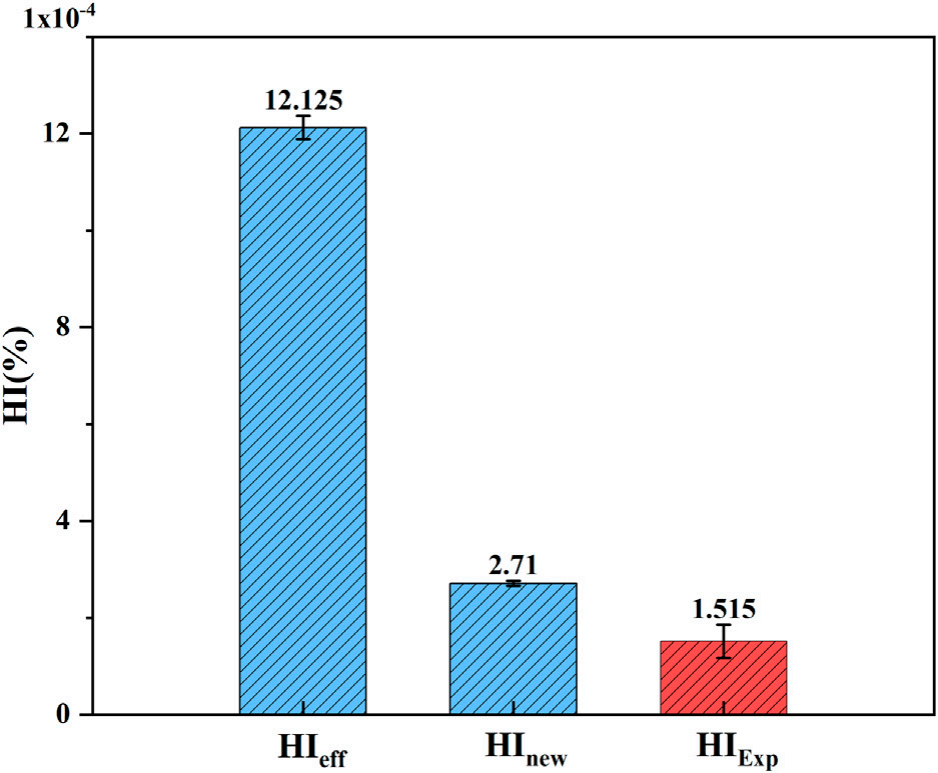
Comparison of hemolysis estimates and experimental values in the axial blood pump.

When a lower shear stress of less than or equal to 25 Pa existed, to which the RBCs were subjected to, the parameter C in the hemolysis models of Eq. 3 and Eq. 7 was corrected by a factor of 0.0416 (Goubergrits, 2006), and then $C=1.5\times 10^{-6}$ . Data on 1,350 traces selected in the magneto-hydraulic suspension pump were separately fed into the parametric corrected hemolysis model, and the hemolysis values of the traces were calculated and averaged to obtain the hemolysis estimation values ${HI}_{eff}$ and ${HI}_{new}$ . Figure 12 shows the comparison between the estimated value of hemolysis and the experimental value of hemolysis ( ${HI}_{\mathrm{Exp}}$ ). The estimated values of the novel hemolysis model proposed in this paper were much closer to the experimental values, which significantly reduced the estimation error. However, there was still a large gap between the estimated and experimental hemolysis values, mainly because the particle trace data could not completely represent the complex flow domain inside the blood pump.

## 4 Conclusion

For blood pumps with a rotating vane structure, the estimation of hemolysis performance is mainly based on a power-law formula, which has a significant estimation error. In this paper, we carried out a study on the equivalent shear stress of RBC aging, analyzed the rationality of RBC aging equivalence in the shear flow field, and developed and validated a novel hemolysis model for blood pumps by considering the initial value of hemolysis and RBC aging equivalence based on the hemolysis model in Eq. 3. Although the estimated hemolysis value of the new model is still slightly different from the experimental hemolysis value, the hemolysis estimation error is effectively reduced compared with the previous hemolysis model, which could provide a more accurate hemolysis model reference for the optimal design of rotary vane blood pumps.

This study also has the limitation that only the effects of RBC aging and initial values of hemolysis on hemolysis estimation were studied. There are other physical factors that can affect hemolysis estimation, such as the temperature factor, were not considered, and many factors that can affect hemolysis estimation should be considered in the blood pump design process.

## Data Availability

The original contributions presented in the study are included in the article/Supplementary Material; further inquiries can be directed to the corresponding authors.
